# Clinical epidemiology and prognostic factors in patients with KPC-producing *K. pneumoniae* infections: a retrospective cohort study

**DOI:** 10.3389/fpubh.2025.1668530

**Published:** 2025-10-24

**Authors:** Qiangsheng Feng, Yuejuan Song, Xiaoqin Ha

**Affiliations:** Department of Clinical Laboratory, The 940th Hospital of Joint Logistics Support Force of People’s Liberation Army, Lanzhou, China

**Keywords:** KPC-Kp, clinical characteristics, survival analysis, mortality, risk factors

## Abstract

**Background:**

This study aimed to investigate the clinical characteristics, drug resistance patterns, and prognosis of CRKP-infected patients.

**Methods:**

This study evaluated in patients with carbapenemase-producing CRKP infection diagnosed through bacteriological evidence and clinical criteria over a 12-month period.

**Results:**

KPC-producing *Klebsiella pneumoniae* represented 1.16% of all *K. pneumoniae* infections, the average patient age was 62.3 ± 20.2 years. Lung infection (58%) was the most common site, followed by bloodstream infection (22%) and urinary tract (11%) infections; 86% were nosocomial. Common comorbidities included cerebrovascular disease/cerebral infarction (23%), lung disease (16%), hematologic diseases/malignancies (12%), and viral pneumonia (12%). KPC-Kp exhibited high resistance (>90%) to most tested antibiotics (including cephalosporins, piperacillin/tazobactam, fluoroquinolones, aztreonam, and carbapenems). Significantly lower resistance was observed only to tigecycline (5.1%) and ceftazidime-avibactam (CAZ-AVI) (4.3%). Non-KPC strains (NDM/VIM/OXA-48; *n* = 48) showed lower resistance (<50%) to several agents and minimal resistance to tigecycline and CAZ-AVI (0–1.0%); resistance differences between KPC and non-KPC groups were highly significant (*p* < 0.001). KPC-Kp infection conferred significantly higher in-hospital mortality (46%) than non-KPC infections (10.4%; *p* < 0.001), with nearly half (48%) of KPC-Kp deaths occurring within 7 days of infection. CAZ-AVI usage within the KPC-Kp group did not significantly improve 28-day survival (0.450 ± 0.132 vs. 0.573 ± 0.076, *p* = 0.317). Multivariate analysis identified significant independent risk factors for in-hospital mortality: KPC-Kp infection (OR 5.96, *p* < 0.001), bloodstream infection (OR 8.57, *p* = 0.006), and ICU admission (OR 3.39, *p* = 0.006).

**Conclusion:**

KPC-Kp infections demonstrated high incidence (1.16%), and severe mortality (46% in-hospital). Mortality risk was significantly elevated by KPC-Kp infection, bloodstream infection, and ICU admission, underscoring critical clinical threats.

## 1 Introduction

*Klebsiella pneumoniae*, a Gram-negative opportunistic pathogen, causes a wide range of community and hospital-acquired infections. Infections caused by carbapenem-resistant *K. pneumoniae* (CRKP) pose a significant public health threat and are strongly associated with high mortality rates, particularly among immunocompromised and critically ill patients (1). Recognizing the severity of this issue, the World Health Organization (WHO) classifies carbapenem-resistant Enterobacteriaceae (CRE), of which CRKP is the most common species, among its highest priority pathogens. Previous studies estimate the pooled mortality rate associated with CRKP infections to range from 33 to 42% (2). Hospital transmission plays a crucial role in CRKP spread; over half of hospitals contributing carbapenemase-positive isolates likely experienced within-hospital transmission, with interhospital spread occurring more frequently within countries than between them (3).

In China, *K. pneumoniae* has become the second most frequently isolated bacterium in clinical settings. Alarmingly, resistance to meropenem has risen steadily from 2.9% in 2005 to 30.0% in 2023.^1^ Furthermore, resistance rates among CRKP isolates exceed 90% for quinolones, *β*-lactams, β-lactam/β-lactamase inhibitor combinations, and aminoglycosides. This escalating resistance prevalence severely limits therapeutic options, intensifying the need for novel strategies to combat CRKP infections (4).

Carbapenem resistance in *K. pneumoniae* is primarily mediated by genes encoding carbapenemases, which are categorized into two main groups: serine-based enzymes (KPC, OXA-48-like, and SME) and metallo-*β*-lactamases (MBLs; including NDM, IMP, and VIM) (5). In this study, we analyzed the impact of different carbapenemase types, antibacterial drug usage patterns, and patient clinical characteristics on treatment outcomes.

## 2 Materials and methods

### 2.1 Study design

① A total of 148 cases of CRKP with pathogenic evidence were included 100 cases KPC-Producing and 48 cases Non- KPC (NDM, VIM, and OXA-48) in this study, with 66% (94/146) ≥2 positive cultures, median number of pathogenic evidence 3 (1, 5) (range 1–94 times), including multi-site infection 24 cases, bloodstream infection 30 cases, lung infection 88 cases, urinary system infection 21cases and, abdominal infection 12 cases.
② A total of 839 rejected for CRKP repeated strains or cases, 20 cases rejected CRKP bacterial colonization, the customization rate is 13.5% (20/148).
③ According to the Clinical and Laboratory Standards Institute (CLSI) guidelines, the definition of carbapenem-resistant *Klebsiella pneumoniae* (CRKP) is primarily based on antimicrobial susceptibility testing results. A strain is classified as CRKP if its minimum inhibitory concentration (MIC) for carbapenem antibiotics—such as imipenem or meropenem—exceeds the specified resistance breakpoint (e.g., MIC >4 mg/L for imipenem or meropenem).

### 2.2 Setting

This study conducted a retrospective review of clinical data from patients diagnosed with CRKP infection disease at the 940th Hospital of the Joint Logistics Support Force of the People’s Liberation Army. Statistical analyses were performed on factors such as antimicrobial therapy (Ceftazidime-Avibactam), enzyme type, infection site, underlying diseases, and in-hospital mortality.

### 2.3 Ethical oversight

The study was approved by the Ethics Committee of the 940th Hospital of the Joint Logistics Support Force of the People’s Liberation Army. The committee waived the need for informed consent. The study adhered to the ethical standards outlined in the Declaration of Helsinki (1975) and its amendments.

### 2.4 Participants

**Figure 1:**
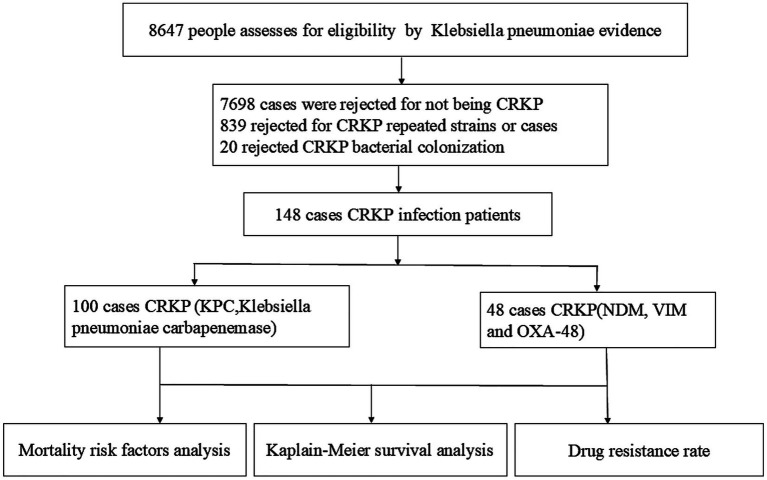
Selection criteria for the inclusion of patients with CRKP.

This study carefully selected participants from 2018 to 2025, ultimately including 148 eligible patients who were followed for 12 months. A detailed flowchart illustrates participant eligibility and reasons for exclusion (Figure 1).

### 2.5 Exposure variables

Demographic and clinical characteristics were analyzed as exposure variables. These included age, sex, KPC-Producing *K. pneumoniae* Infections, antimicrobial therapy (Ceftazidime -Avibactam, sensitive for antimicrobial sensitivity test), infection site, ICU admission, median hospital time (20 day), Pathogen source.

### 2.6 Endpoint

The primary endpoint was in-hospital mortality among CRKP inpatients for 12 month.

### 2.7 *K. pneumoniae* culture, identification, and antimicrobial susceptibility test

All the subjects recorded episodes who were hospitalized and suspected of BSIs (Bloodstream infection) between January 2018 and January 2025, blood cultures were obtained using BacT/ALERT blood culture bottles (bio-Mérieux, Inc., Durham, NC) or BD FA and SN blood culture bottles and incubated in the BacT/ALERT 3D (bioMérieux, Inc.) or BD FX 400 automatic monitoring system for a week in the clinical microbiology laboratory of the hospital. When Bottles flagged as positive after Gram-negative Bacillus, report the critical value and switch to blood culture onto blood tablet and Chinese blue agar plates and incubated at 35°C CO_2_ for 24 h. BALF and qualified sputum specimens were also inoculated onto blood tablet and Chinese blue agar plates and incubated at 35°C CO_2_ for 24 h. It is clinically significant that the count value of urine colonies Midstream urine culture is ≥ 1 × 105 cfu/mL, so colony identification and antimicrobial susceptibility test should be carried out. After colony formation, microbial identification was performed using the corresponding GN card on the VITEK Compact-II automatic microorganism identification system or MALDI-TOF MS. Antimicrobial susceptibility test was used GN 335 card in VITEK 2 system (bioMérieux, Inc., Durham, NC). A strain is classified as CRKP if its minimum inhibitory concentration (MIC) for carbapenem antibiotics—such as imipenem or meropenem—exceeds the specified resistance breakpoint (e.g., MIC >4 mg/L for imipenem or meropenem).

### 2.8 Enzyme type detection

Modified Carbapenem Inactivation Methods (mCIM): For each isolate, emulsify a 1-μL loopful (*K. pneumoniae*) from a blood agar plate into 2 mL TSB. Vortex 10–15 s, add a 10-μg meropenem disk, ensuring full immersion. Incubate at 35°C ± 2°C for 4 h ± 15 min. Concurrently, prepare a 0.5 McFarland *E. coli* ATCC^®^ 25922 suspension in broth/saline. Inoculate an MHA plate per CLSI M024 (6), completing suspension prep and plate inoculation within 15 min. Dry plates 3–10 min. Post-incubation, remove meropenem disks from TSB using a 10-μL loop: press the loop’s flat side against the disk edge, leveraging surface tension to lift it. Drain excess liquid by dragging the loop against the tube’s inner edge, then transfer the disk to the inoculated MHA plate (max 4 disks/100-mm plate; 8/150-mm plate). Invert and incubate plates at 35°C ± 2 C for 18–24 h. Measure inhibition zones per CLSI M024, Carbapenemase positive: Zone diameter of 6–15 mm or presence of pinpoint colonies within a 16–18-mm zone. Metallo-*β*-lactamase positive: ≥5-mm increase in zone diameter for eCIM vs. zone diameter for mCIM (e.g., mCIM = 6 mm; eCIM = 15 mm; zone diameter difference = 9 mm) (6). Ensure timing alignment: *E. coli* suspension prep and plate inoculation must occur ≤15 min before/after TSB-disk incubation completion. Maintain aseptic technique throughout. Colloidal gold method for determination of CRKP: Pick the pure cultured colony of *K. pneumoniae*, add the lysate and shake it evenly, and drop the lysate into the sample adding hole of colloidal gold test strip (Jinshanchuan, Beijing). Observe the results within 10–15 min, and the T line and the quality control line (C line) are positive at the same time (specific enzyme type: KPC, NDM, IPM, VIM, and OXA-48), Only C-ray was negative.

### 2.9 Clinical report

Laboratory-confirmed CRKP, report to the clinical department immediately as a critical value and enzyme type, communicated by telephone. Implement single-room isolation and dedicated care. If associated with an indwelling venous or catheter device, remove and replace the device promptly.

### 2.10 Statistical analyses

Statistical analyses were performed using SPSS 22.0. A paired *t*-test revealed significantly higher overall drug resistance in the KPC group compared to non-KPC isolates. Multivariate regression identified the following independent predictors of mortality: KPC-Kp infection, ICU admission, bloodstream infection, specific antimicrobial therapy, nosocomial acquisition, and prolonged hospitalization (≥20 days). The log-rank test showed significantly lower 28-day survival in KPC-Kp patients versus non-KPC groups, particularly between Ceftazidime-Avibactam and non-Ceftazidime-Avibactam subgroups. Kaplan–Meier survival curves were constructed with the time of first etiological diagnosis as the starting point, death as the event of interest, and discharge as censoring. Factors associated with in-hospital mortality were visualized using a forest plot (*p* < 0.01). The significance threshold was set at *p* < 0.05 for all analyses.

## 3 Results

### 3.1 Clinical features of patients with KPC-producing *K. pneumoniae* infections

A cohort of 100 patients with KPC-producing *K. pneumoniae* (KPC-Kp) infections was analyzed, representing 67.6% of all CRKP isolates (KPC: 100 cases; NDM, VIM, OXA-48: 48 cases). The mean patient age was 62.3 ± 20.2 years, with a male-to-female ratio of 1.8:1. KPC-producing *Klebsiella pneumoniae* represented 1.16% of all *K. pneumoniae* infections (100/8647). Twenty-eight percent of patients had hospital stays exceeding 14 days, with a median duration of 58 days (IQR 30–165). The predominant infection sites were lung (58%), bloodstream (22%), and urinary tract (11%). Pathogen sources were primarily nosocomial (86%), with 14% with 14% transfer from outside hospital, colonization rate 13.5% (20/148). Major comorbidities included cerebrovascular disease/cerebral infarction (23%), pulmonary disease (16%), hematological malignancies (12%), and viral pneumonia (12%) (Table 1).

**Table 1:** Host factor in PC-producing *K. pneumoniae* infections (*n* = 100 case).

Host factors	Cases	%
Hematological diseases and malignant tumors	12	12
Cerebrovascular disease and cerebral infarction	23	23
Lung infection	16	16
Viral pneumonia	12	12
Severe acute pancreatitis	4	4
Multiple injuries	6	6
Abdominal infection	6	6
Diabetes	5	5
Others	16	16

### 3.2 CRKP drug resistance rates

**Figure 2:**
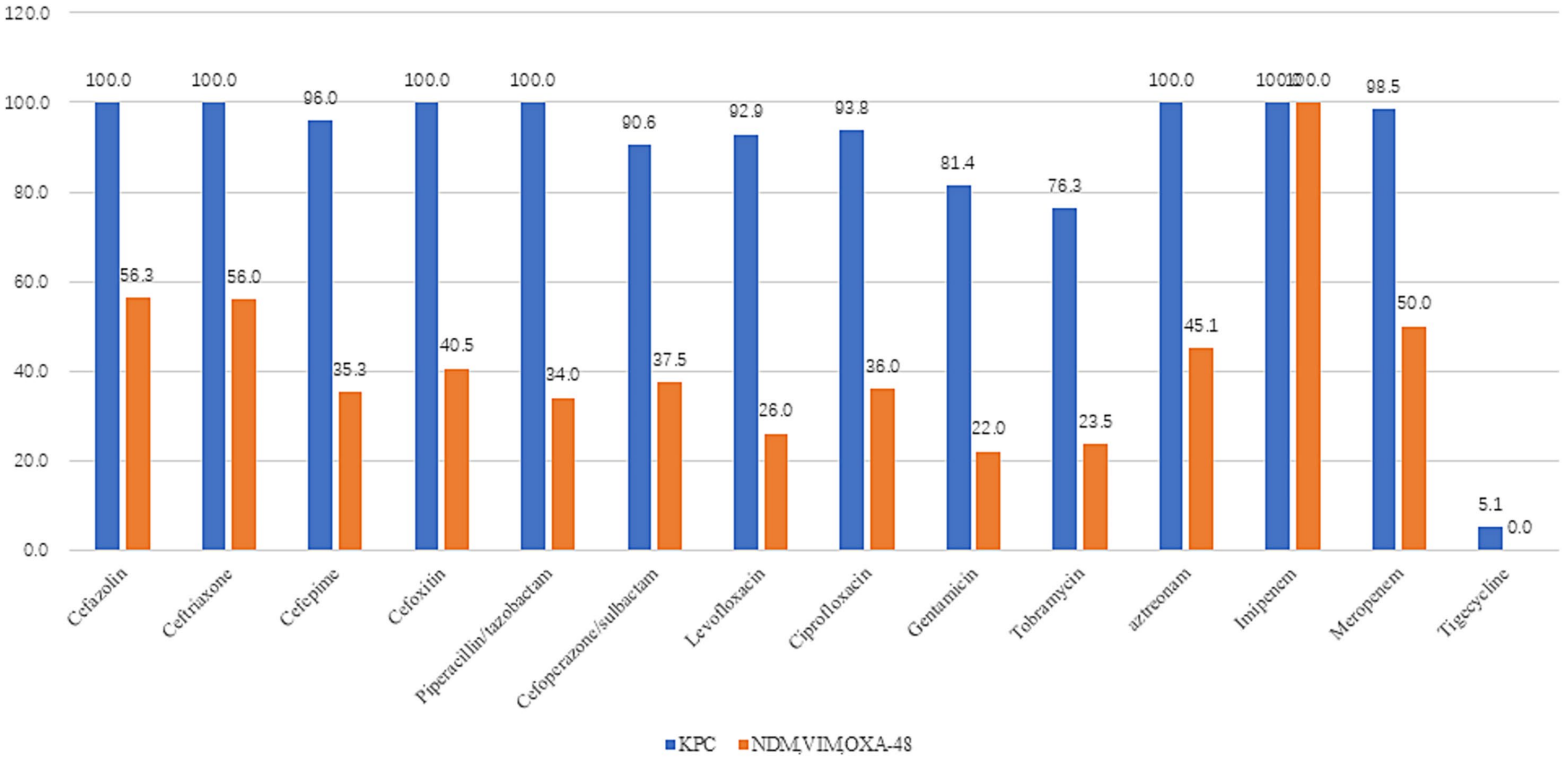
Drug resistance rates of different enzyme types in *Klebsiella pneumoniae* (KPC = 100cases, NDM, VIM, and OXA-48 = 48case). KPC producers exhibited >90% resistance to cefazolin, ceftriaxone, cefepime, cefoxitin, piperacillin/tazobactam, cefoperazone/sulbactam, levofloxacin, ciprofloxacin, aztreonam, imipenem, and meropenem, while demonstrating markedly lower resistance to tigecycline (5.1%) and ceftazidime/avibactam (4.3%). In contrast, non-KPC strains (NDM/VIM/OXA-48) showed <50% resistance to cefepime, cefoxitin, piperacillin/tazobactam, cefoperazone/sulbactam, levofloxacin, ciprofloxacin, and aztreonam, with near-zero resistance to tigecycline (0%) and ceftazidime/avibactam (1.0%). Paired t-test confirmed significantly higher overall resistance in KPC versus non-KPC groups (*t* = 7.617, *p* < 0.001).

Analysis of 148 CRKP isolates revealed KPC-producing *K. pneumoniae* (67.6%, *n* = 100) as the predominant strain, followed by NDM (23.6%, *n* = 35), VIM (3.4%, *n* = 5), and OXA-48 (5.4%, *n* = 8). KPC producers exhibited >90% resistance to cefazolin, ceftriaxone, cefepime, cefoxitin, piperacillin/tazobactam, cefoperazone/sulbactam, levofloxacin, ciprofloxacin, aztreonam, imipenem, and meropenem, while demonstrating markedly lower resistance to tigecycline (5.1%) and ceftazidime-avibactam (4.3%). In contrast, non-KPC strains (NDM/VIM/OXA-48) showed <50% resistance to cefepime, cefoxitin, piperacillin/tazobactam, cefoperazone/sulbactam, levofloxacin, ciprofloxacin, and aztreonam, with near-zero resistance to tigecycline (0%) and ceftazidime-avibactam (1.0%). Paired *t*-test confirmed significantly higher overall resistance in KPC versus non-KPC groups (*t* = 7.617, *p* < 0.001) (Figure 2).

### 3.3 Survival outcomes in KPC-producing *K. pneumoniae* infections

**Figure 3:**
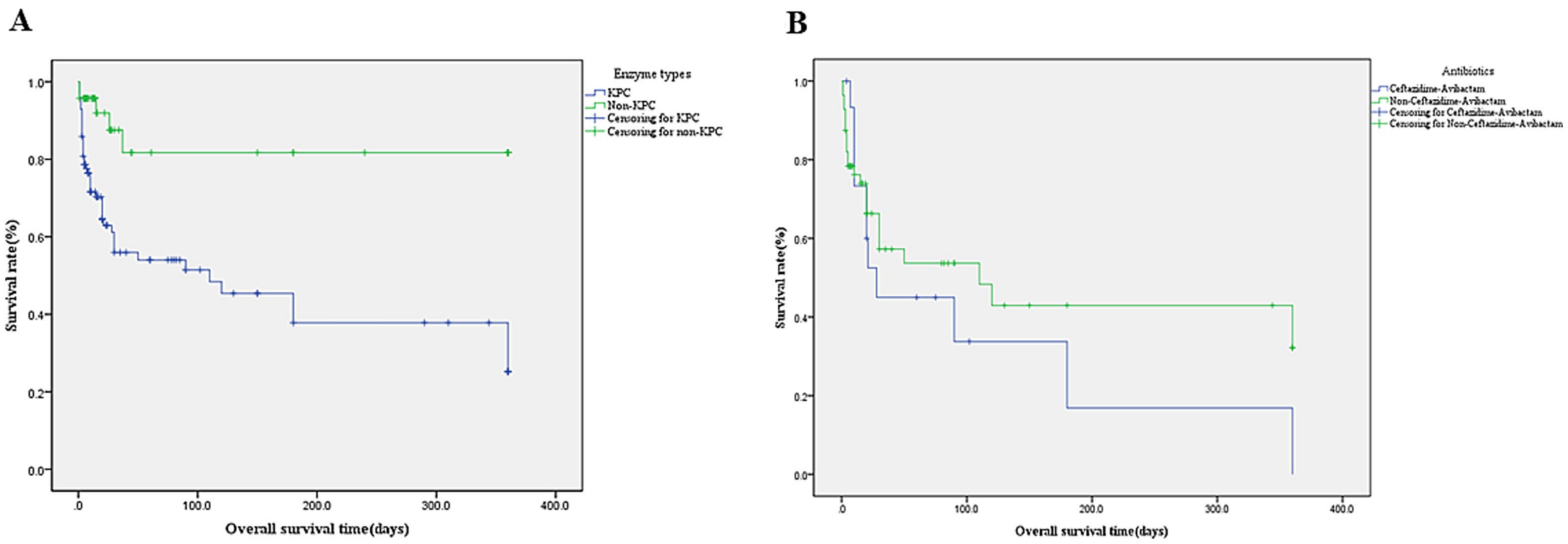
**(A)** Kaplan–Meier survival curve of enzymetype. KPC-producing vs. Non- KPC-producing mean survival days (164.1 ± 21.2) vs. (286.6 ± 28.3) and 28 days survival rate 0.609 ± 0.054 vs. 0.839 ± 0.069, χ=17.732, *p* < 0.001. **(B)** Kaplan–Meier survival curve of Ceftazidime-Avibactam (*n* = 16 cases). Ceftazidime-Avibactam vs. Non-Ceftazidime-Avibactam Mean survival days (110.1 ± 41.0) vs. (174.2 ± 29.1),28 days survival rate 0.450 ± 0.132 vs. 0.573 ± 0.076, χ=1.001, *p* = 0.317.

Among 100 patients with KPC-Kp infections, the in-hospital mortality rate was 46.0%—significantly higher than the 10.4% mortality in non-KPC carbapenemase producers (NDM/VIM/OXA-48; *p* < 0.001), with nearly half (48%) of KPC-Kp deaths occurring within 7 days of infection. Survival analysis demonstrated substantially lower 28-day survival in KPC-Kp patients (0.609 ± 0.054) versus non-KPC groups (0.839 ± 0.069; log-rank χ^2^ = 17.732, *p* < 0.001), with KPC-Kp 90-day survival at 0.514 ± 0.061. Within the KPC-Kp cohort, 28-day survival did not significantly differ between patients receiving ceftazidime-avibactam (0.450 ± 0.132) and those not receiving this agent (0.573 ± 0.076; *χ*^2^ = 1.001, *p* = 0.317) (Figures 3A,B). KPC vs. NDM, VIM, OXA-48 28 days survival rate 0.609 ± 0.054 vs. 0.839 ± 0.069, *χ* = 17.732, *p* < 0.001, KPC-Kp infections demonstrated 5-day and 90-day survival rates of 0.769 ± 0.042 and 0.514 ± 0.061, respectively. In KPC-Producing K. pneumoniae infections patients, the usage rate of Ceftazidime-Avibactam vs. Non- Ceftazidime-Avibactam 28 days survival rate 0.450 ± 0.132 vs. 0.573 ± 0.076, χ = 1.001, *p* = 0.317.

### 3.4 Risk factors associated with KPC-producing *K. pneumoniae* infections patient in-hospital death

**Figure 4:**
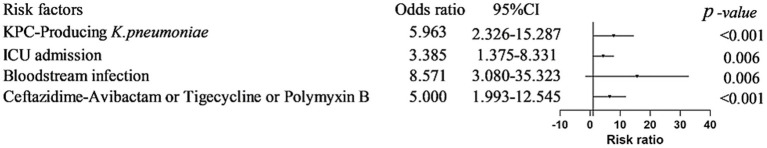
Risk factors associated with KPC-KP patient in-hospital death. KPC infection (OR 5.96, 95%CI 2.33–15.29, *p* < 0.001), bloodstream infection (OR 8.57, 95%CI 3.08–23.85, *p* = 0.006), ICU admission (OR 3.39, 95%CI 1.38–8.33, *p* = 0.006), Antimicrobial agents (ceftazidime/avibactam, tigecycline or polymyxin B; OR 5.00, 95%CI 1.74–14.37, *p* < 0.001).

Univariate analysis identified KPC-KP infection, ICU admission, infection site (particularly bloodstream), antimicrobial therapy, nosocomial acquisition, and prolonged hospitalization (median ≥20 days) as mortality-associated factors. Multivariate regression confirmed independent mortality predictors: KPC infection (OR 5.96, 95%CI 2.33–15.29, *p* < 0.001), bloodstream infection (OR 8.57, 95%CI 3.08–23.85, *p* = 0.006), ICU admission (OR 3.39, 95%CI 1.38–8.33, *p* = 0.006), Susceptibility-guided antimicrobial therapy (ceftazidime/avibactam, tigecycline or polymyxin B; OR 5.00, 95%CI 1.74–14.37, *p* < 0.001) (Figures 3, 4 and Table 2).

**Table 2:** Demographic, clinical and laboratory, findings of patients on admission.

Demographics and clinical characteristics	Total (*n* = 100 cases)	Nonsurvivor	Survivor	*P*
		(*n* = 54 cases)	(*n* = 46 cases)	
Sex				*p* = 0.283
Female	36 (42%)	22 (61%)	14 (39%)	
Male	64 (58%)	32 (50%)	32 (50%)	
Age				*p* = 0.135
≥60	55 (55%)	29 (53%)	26 (47%)	
<60	45 (45%)	17 (38%%)	28 (62%)	
KPC-producing *K. pneumoniae* infections				*p* < 0.001
Yes	100 (68%)	46 (46%)	54 (54%)	
No	48 (32%)	6 (13%)	42 (87%)	
ICU admission				*p* = 0.006
Yes	30 (30%)	20 (66%)	10 (33%)	
No	70 (70%)	26 (37%)	44 (63%)	
Infection site				*p* = 0.006
Lung infection	58 (58%)	27 (47%)	31 (53%)	
Bloodstream infection	22 (22%)	15 (68%)	7 (32%)	
Others (urinary = 11cases)	20 (20%)	4 (20%)	16 (80%)	
Median hospital time = 20 day				*p* = 0.044
≥20 day	50 (50%)	16 (32%)	32 (68%)	
<20 day	50 (50%)	28 (56%)	22 (44%)	
Antimicrobial agents				*p* = 0.003
Cefotaxime/Avibactam	16 (16%)	11 (69%)	5 (31%)	
Tigecycline or polymyxin B	16 (16%)	12 (75%)	4 (25%)	
IPM or MEN*	40 (40%)	12 (30%)	28 (70%)	
No antibacterial drugs used	28 (28%)	11 (39%)	17 (61%)	
Pathogen source				*p* = 0.401
Nosocomial infection	86 (86%)	41 (48%)	45 (52%)	
Transfer from outside hospital	14 (14%)	5 (36%)	9 (64%)	

*Meropenem 13cases, Cefoperazone/sulbactam 14 case, Piperacillin/tazobactam 4 cases, Imipenem 2 cases. Data are median (IQR) or n (%). *p*-values were calculated by Mann–Whitney U test, χ^2^ test, or Fisher’s exact test, as appropriate.

## 4 Discussion

Carbapenem-resistant *Klebsiella pneumoniae* (CRKP) is the most prevalent carbapenem-resistant species, with *K. pneumoniae* carbapenemase (KPC) serving as the predominant carbapenemase (7). In our study, the majority (86%) of infections were nosocomial, while only 14% originated from external healthcare facilities. A colonization rate of 13.5% (20/148) underscores the need for multifaceted infection control interventions to curb colonization and cross-transmission (8). At our institution, KPC-producing *K. pneumoniae* (KPC-Kp) accounted for 1.16% (100/8647) of all *K. pneumoniae* infections—lower than the rate reported by Hu et al. based on 2023 CHINET data (4). In contrast, meropenem resistance reached 30.0% in 2023. These findings demonstrate that laboratory detection of clinical isolates and infection control practices critically influence CRKP prevalence in hospitals.

In our cohort, the predominant infection sites were the lung (58%), followed by bloodstream (22%), and urinary tract (11%). This distribution differs significantly from published data reporting bloodstream infections (50.1%), lower respiratory tract infections (33.3%), and complicated urinary tract infections (8.8%) (9). Major comorbidities included cerebrovascular disease/cerebral infarction (23%), pulmonary disease (16%), hematological malignancies (12%), and viral pneumonia (12%), primarily affecting patients with pulmonary involvement or extended hospital stays. Notably, older adults patients with severe comorbidities often require tracheal intubation (10), suggesting potential emergence of carbapenem-resistant hypervirulent *K. pneumoniae* (CR-hvKP) in this high-risk population.

Among 148 CRKP isolates in our study, KPC-producing *K. pneumoniae* (KPC-Kp) was predominant (67.6%, *n* = 100), followed by NDM (23.6%, *n* = 35), OXA-48 (5.4%, *n* = 8), and VIM (3.4%, *n* = 5). This distribution aligns with reported KPC dominance (77%) in Chinese CRKP isolates from 2012 to 2016 (11). KPC-Kp strains exhibited >90% resistance to most antibiotics tested—including penicillins (piperacillin/tazobactam), cephalosporins (cefazolin, ceftriaxone, cefepime, cefoxitin, cefoperazone/sulbactam), fluoroquinolones (levofloxacin, ciprofloxacin), aztreonam, and carbapenems (imipenem, meropenem)—but showed markedly lower resistance to tigecycline (5.1%) and ceftazidime/avibactam (4.3%). In contrast, non-KPC strains (NDM/VIM/OXA-48) demonstrated <50% resistance to these same agents (excluding carbapenems) and near-complete susceptibility to tigecycline (0% resistance) and ceftazidime/avibactam (1.0% resistance). Consistent with previous studies, KPC-KP exhibited higher resistance rates to antibiotics than NDM-KP (12, 13). A paired *t*-test confirmed significantly higher overall resistance in KPC versus non-KPC strains (*t* = 7.617, *p* < 0.001), underscoring the necessity for enhanced clinical vigilance and institution-specific infection control protocols against KPC-Kp (14).

In our study of 100 patients with KPC-Kp infections, the in-hospital mortality rate was 46.0%—significantly higher than the 10.4% mortality observed in non-KPC carbapenemase producers (NDM/VIM/OXA-48; *p* < 0.001). Nearly half (48%) of KPC-Kp deaths occurred within 7 days of infection. This mortality rate exceeds the pooled rate of 33% reported in a recent meta-analysis of KPC-producing CRKP infections (21 studies across seven countries, 2007–2018) (15), and is also higher than the literature-reported 30-day mortality rate of 31.6% (108/342) for KPC-Kp infections.

Survival analysis demonstrated significantly lower 28-day survival in KPC-Kp patients (0.609 ± 0.054) versus non-KPC groups (0.839 ± 0.069; log-rank χ^2^ = 17.732, *p* < 0.001), with 90-day survival at 0.514 ± 0.061 for KPC-Kp. This disparity may be attributed to higher antibiotic resistance in KPC strains and fewer therapeutic options compared to non-KPC CRKP. Within the KPC-Kp cohort, 28-day survival showed no significant difference between patients receiving ceftazidime-avibactam (0.450 ± 0.132) and those not receiving this agent (0.573 ± 0.076; χ^2^ = 1.001, *p* = 0.317). However, literature reports indicate mortality rates of 18.3% (16), 23.4% (17), 25% (18), 28.1% (19), and 34% (20) for ceftazidime-avibactam treatment of KPC-Kp infections, collectively suggesting suboptimal efficacy.

Multivariate regression confirmed independent mortality predictors: KPC-Kp infection (OR 5.96, 95%CI 2.33–15.29, *p* < 0.001), KPC-Kp bloodstream infection (OR 8.57, 95%CI 3.08–23.85, *p* = 0.006), ICU admission (OR 3.39, 95%CI 1.38–8.33, *p* = 0.006), and necessity of reserve antimicrobials (ceftazidime/avibactam, tigecycline, or polymyxin B; OR 5.00, 95%CI 1.74–14.37, *p* < 0.001). This aligns with literature demonstrating that risk factor analysis for CRKP bloodstream infections (BSIs) and their association with 28-day mortality enhances clinical understanding of BSI pathogens (21, 22), while ICU admission specifically correlates with KPC-Kp colonization (12.8%, 52/405), where prior ICU stay constitutes a major risk factor (OR 12.5, 95%CI 1.8–86.8) (23). Furthermore, pooled mortality analysis reveals temporally escalating odds ratios for CRKP versus non-CRKP infections: 7-day OR 3.22 (95%CI 1.18–8.76), 14-day OR 5.66 (95%CI 4.31–7.42), 28/30-day OR 3.87 (95%CI 3.01–4.98), and in-hospital OR 4.05 (95%CI 3.38–4.85) (24).

Conclusion, KPC-producing KP dominated (67.6%) in this study, exhibiting extensive antibiotic resistance (>90% to most agents). KPC infections caused significantly higher in-hospital mortality (46.0%) than non-KPC strains (10.4%), with 48% of KPC deaths occurring within 7 days. Mortality predictors included KPC infection itself (OR 5.96), KPC bloodstream infection (OR 8.57), ICU admission (OR 3.39), and needing reserve antibiotics (OR 5.00). This underscores urgent need for enhanced control and treatment strategies.

## 5 Limitation

Due to economic reasons, the number of people receiving KPC-KP Ceftazidime-Avibactam treatment is small. The major limitation of this study is that the enzymatic detection results were not confirmed by genetic amplification (e.g., PCR), which could lead to potential false-positive or false-negative results. The single-center design of this study may restrict the generalizability of its findings to other healthcare settings or geographic regions.

## Data Availability

The raw data supporting the conclusions of this article will be made available by the authors, without undue reservation.

## Footnotes

^1^ http://www.chinets.com/Data/GermYear
